# Radical Resection of a Giant Facial Plexiform Neurofibroma in a Resource-Limited Setting

**DOI:** 10.1177/22925503261485189

**Published:** 2026-09-11

**Authors:** Tertius Venter, Alan D. Rogers, Konrad Mende, Thandiwe Munaiwa, Andrew Hodges

**Affiliations:** 1 Plastic and Reconstructive Surgery, 701261 Mercy Ships, Garden Valley, TX, USA; 2 Department of Surgery, 483372 University of Toronto, Toronto, ON, Canada; 3 Ross Tilley Burn Centre, Sunnybrook Health Sciences Centre, Toronto, ON, Canada; 4 Department of Orthopaedics, 388063 Sonnenhof, Bern, Switzerland; 5 248473 Parirenyatwa Hospital, Harare, Zimbabwe; 6 CURE Zimbabwe, Bulawayo, Zimbabwe

**Keywords:** Anterolateral thigh flap, facial neoplasms, global surgery, neurofibromatosis 1, plexiform neurofibroma, reconstructive surgical procedures, lambeau antérolatéral de cuisse, néoplasmes faciaux, chirurgie mondiale, neurofibromatose de type 1, neurofibrome plexiforme, procédures de chirurgie reconstructive

## Abstract

Giant facial plexiform neurofibromas (PNFs) in neurofibromatosis type 1 are surgically challenging, particularly in low- and middle-income countries where patients may present late and repeated staged procedures may be impractical. We report a 20-year-old woman from rural Zimbabwe with a massive recurrent left facial PNF after three prior debulking procedures. She underwent single-stage radical excision of involved skin, mucosa, parotid tissue, and facial nerve branches, followed by immediate anterolateral thigh free flap reconstruction and static facial suspension. Two units of packed red blood cells were transfused, the flap survived, and she was discharged on postoperative day 6. At 22 months, a small localized mucosal recurrence had been excised without further recurrence. This case, considered alongside a 34-patient operative experience, supports definitive macroscopic clearance with immediate reconstruction as a pragmatic strategy when repeated debulking is unlikely to provide durable benefit.

## Graphical abstract

**Graphical abstract:**
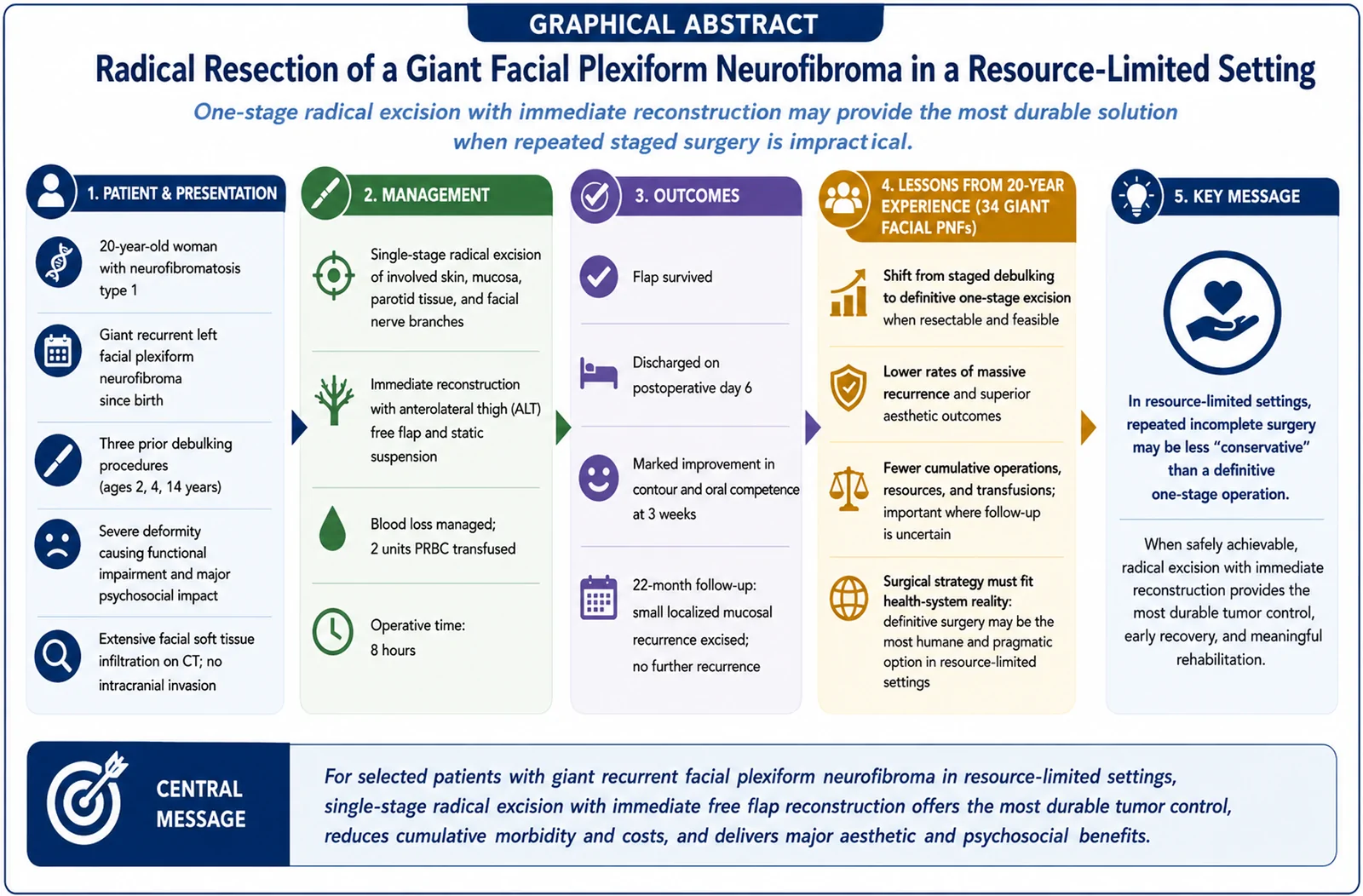
This is a visual representation of the abstract.

## Résumé

Les neurofibromes plexiformes géants de la face associés à la neurofibromatose de type 1 représentent un défi chirurgical majeur, particulièrement dans les milieux à ressources limitées où les patients se présentent souvent tardivement et où les interventions répétées sont difficiles à assurer. Nous rapportons le cas d’une femme de 20 ans originaire du Zimbabwe présentant un neurofibrome plexiforme géant récidivant de l’hémiface gauche après trois réductions tumorales antérieures. Elle a bénéficié d’une exérèse radicale en un seul temps des tissus atteints, incluant la peau, la muqueuse, le tissu parotidien et des branches du nerf facial, suivie d’une reconstruction immédiate par lambeau libre antérolatéral de cuisse et suspension statique. Le lambeau a survécu et la patiente est sortie au sixième jour postopératoire. À 22 mois, une petite récidive muqueuse localisée avait été excisée sans autre récidive. Ce cas, éclairé par une expérience de 34 patients, soutient une stratégie d’exérèse macroscopique définitive lorsque des chirurgies incomplètes répétées sont peu susceptibles d’apporter un bénéfice durable.

## Introduction

Plexiform neurofibromas (PNFs) are benign but infiltrative peripheral nerve sheath tumors associated with neurofibromatosis type 1 (NF1).^1^ When they involve the face and reach giant proportions, they may cause visual loss, oral incompetence, major disfigurement, psychosocial stigma, and recurrent growth after incomplete excision.^2,3^ Key clinical, imaging, and management considerations for giant PNFs are summarized in Table 1. Management is especially difficult in low- and middle-income countries (LMICs), where patients often present late and where access to imaging, blood products, intensive care, microsurgery, and serial reconstructive procedures may be limited^4^ This case illustrates a definitive single-stage strategy for a recurrent giant facial PNF treated in Zimbabwe, informed by the senior author's 20-year experience managing 34 similar patients in resource-limited environments.

**Table 1.** Summary of the Epidemiology, Pathology, Clinical Features, Imaging Findings, Complications, Management Strategies, and Prognosis of Giant Plexiform Neurofibromas.

Domain	Details
Epidemiology	- Affect up to 30% of patients with neurofibromatosis type 1 (NF1). - Giant PNFs (≥5 cm or involving an entire anatomical subunit) are uncommon, but most often seen in the head and neck. - Typically present in childhood; growth accelerates during puberty.
Pathology	- Benign peripheral nerve sheath tumors composed of Schwann cells, fibroblasts, perineural cells, and axons. - Characterized by diffuse, infiltrative growth along multiple nerve fascicles, extending into skin, subcutaneous tissue, muscle, and bone.
Natural History	- Progressive enlargement with recurrent growth after subtotal resections. - May cause major craniofacial disfigurement, functional impairment, and psychosocial stigma. - Risk of malignant transformation into malignant peripheral nerve sheath tumor (MPNST): 10%−15% lifetime risk.
Clinical Features	- Soft, often pendulous mass with “bag of worms” consistency. - Disfigurement: hemifacial hypertrophy, ptosis, ectropion, oral incompetence. - Functional compromise: visual loss, airway obstruction, mastication difficulty, speech impairment. - Neurological: cranial nerve palsy from infiltration or prior surgery.
Imaging	- MRI: gold standard; T2 hyperintensity with a multilobulated, infiltrative “bag of worms” appearance. Defines soft-tissue and perineural spread. - CT: demonstrates bony dysplasia (sphenoid wing absence, orbital enlargement, mandibular or zygomatic hypoplasia). - Essential for preoperative planning, resection margins, and reconstructive strategy.
Complications	- Severe intraoperative haemorrhage (up to 40 units transfusion reported). - Difficult wound healing due to poor tissue quality. - High recurrence after incomplete excision (40%−60%). - Progressive psychosocial morbidity if untreated.
Surgical Approaches	- Staged debulking: palliative, preserves function but almost invariably followed by regrowth; cumulative scarring complicates re-operations. - Radical excision: en bloc resection of all involved tissue, including nerves, parotid, and overlying skin if necessary. Offers best long-term control but at cost of functional sacrifice. - Reconstruction: microvascular free flaps (ALT, latissimus dorsi) restore contour and bulk; suspension sutures or static slings counteract gravitational ptosis.
Adjuncts to Surgery	- Preoperative epinephrine infiltration and electrocautery to reduce bleeding. - Multidisciplinary planning (plastic, neurosurgery, maxillofacial, anaesthesia). - ICU/postoperative monitoring critical for major resections.
Non-Surgical Approaches	- Supportive: prosthetics, psychosocial support, rehabilitative therapy. - Pharmacologic: MEK inhibitors (e.g., selumetinib) reduce tumor burden in inoperable paediatric PNFs; limited global availability
Prognosis	- Radical excision with reconstruction provides best chance for long-term tumor control and improved cosmesis. - Subtotal excision associated with high recurrence and repeated operations. - Quality of life improves substantially with reduction of disfigurement, even if nerve sacrifice is required.

Case Report

**Figure 1.**
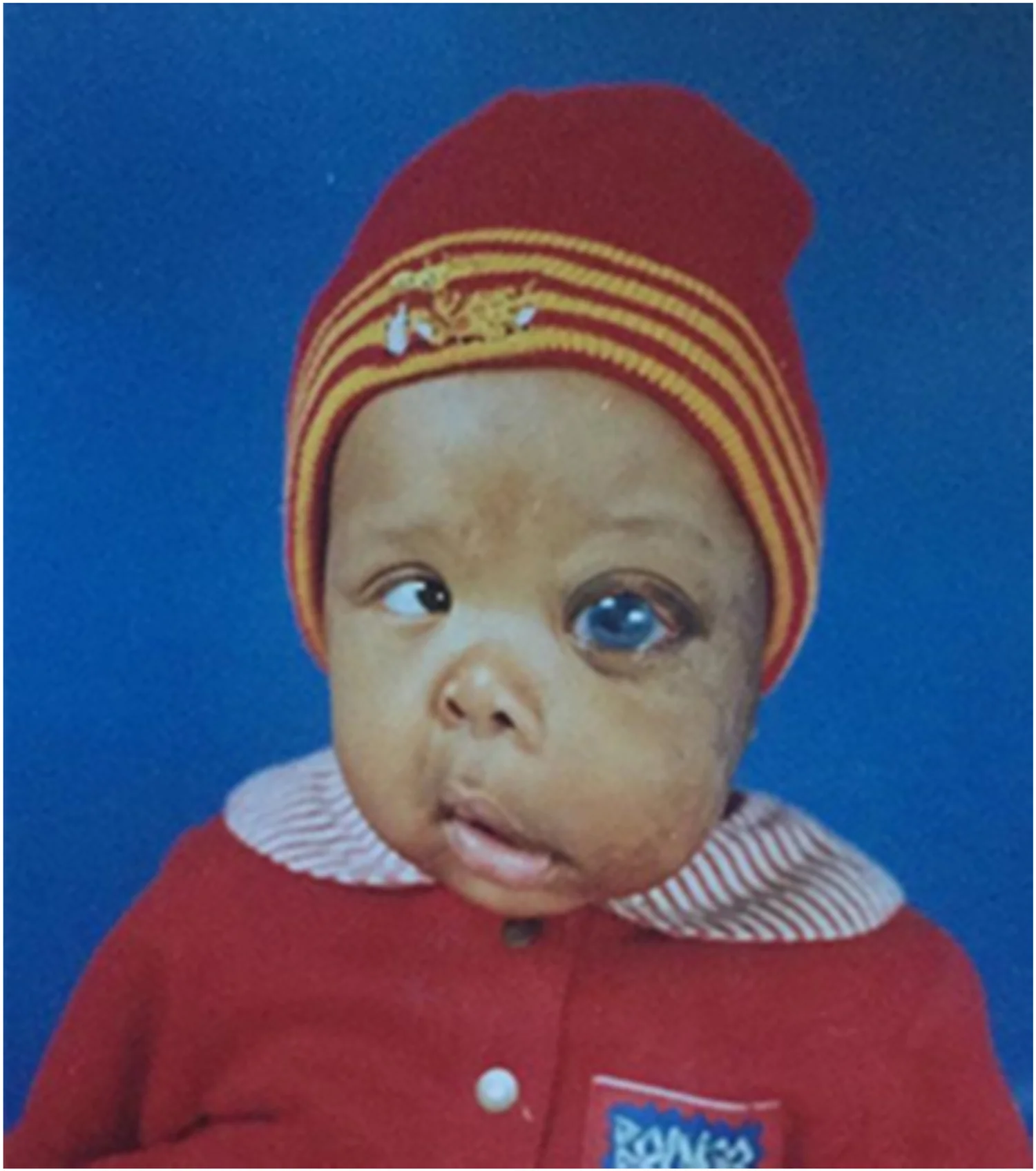
Appearance at age 2, showing a left orbital prosthesis and soft tissue swelling of the left cheek due to giant facial plexiform neurofibroma.

**Figure 2.**
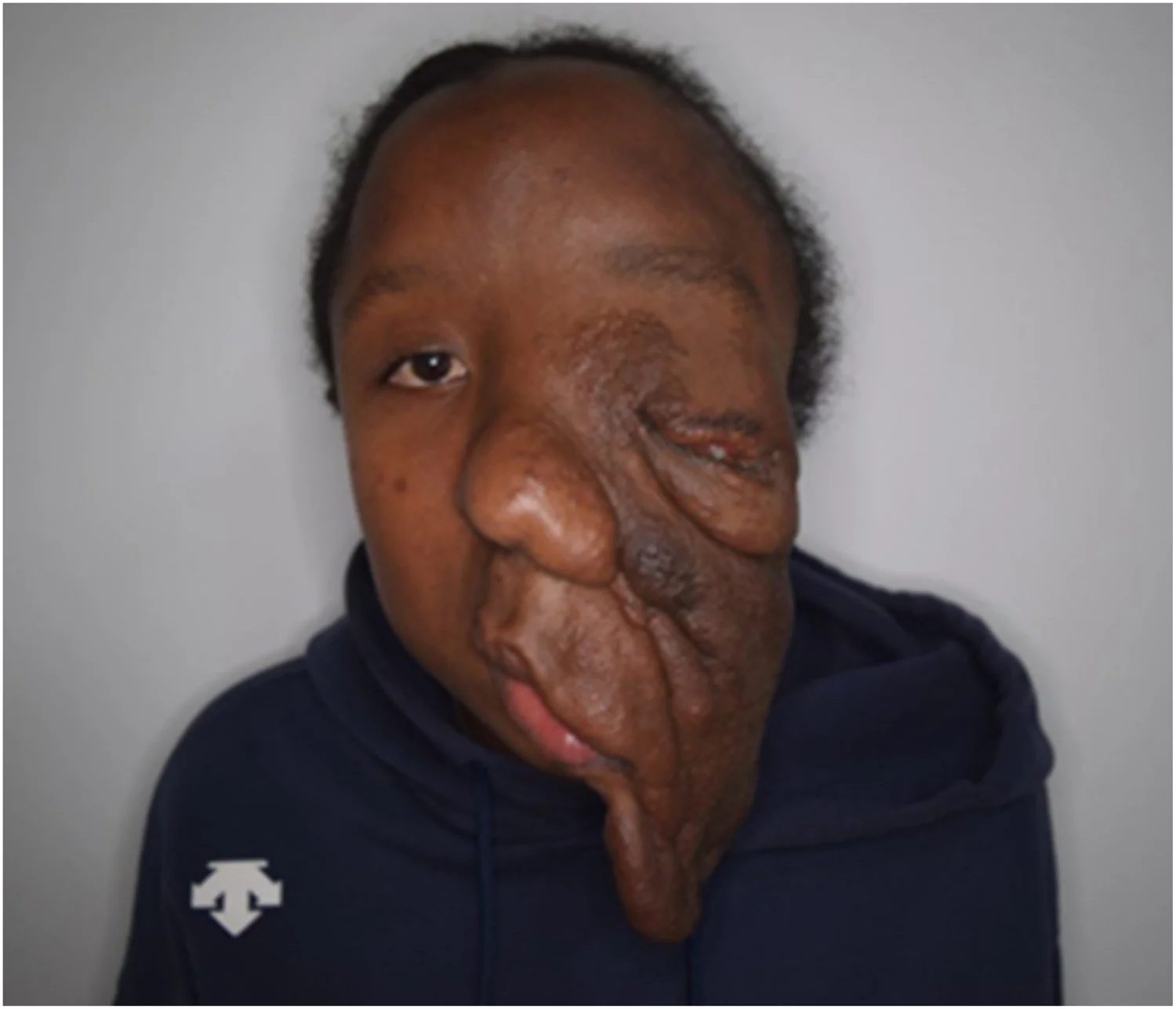
Preoperative appearance in late childhood after two prior debulking procedures performed in a resource-limited setting, with persistent midface distortion.

A 20-year-old woman from rural Zimbabwe with NF1 presented with a massive recurrent left facial PNF. A subtle periorbital swelling had been present at birth. By age 2 years, glaucoma and blindness led to left eye enucleation and initial peri-orbital tumor debulking (Figure 1). Further debulking procedures at ages 4 and 14 years were followed by recurrent growth, progressive cheek ptosis, and persistent hemifacial distortion (Figure 2). Planned revision in 2019 was deferred during the COVID-19 pandemic. She reported heaviness and cosmetic deformity, but no pain, ulceration, bleeding, airway symptoms, or cognitive impairment.^4^

Examination showed a pendulous left hemifacial tumor extending from the brow and upper eyelid region to the mandibular border, medially to the nose and upper lip, and laterally to the preauricular region. The lesion involved thickened skin and was palpable through the buccal mucosa. She had no vision in the left eye and pre-existing left facial nerve weakness, but maintained oral competence. Computed tomography demonstrated diffuse left facial soft-tissue infiltration, sphenoid wing dysplasia, zygomatic and maxillary hypoplasia, mandibular and temporal bone atrophy, and extension into the orbit and maxillary sinus without intracranial invasion.

Because three subtotal operations had failed, a definitive single-stage resection and reconstruction was planned. Surgery was undertaken at a charitable surgical hospital with limited but functional reconstructive capacity. The team prepared for haemorrhage with blood availability, epinephrine infiltration, tranexamic acid, careful anaesthetic monitoring, and two senior surgeons operating together.

Resection incorporated prior scars and removed all grossly abnormal skin, mucosa, parotid tissue, and tumor-infiltrated soft tissue. Approximately 5 × 5 cm of involved buccal mucosa and muscle was excised. Grossly involved zygomatic and buccal facial nerve branches were sacrificed, as preservation would have increased operative time, bleeding, and residual disease in a nerve already clinically impaired. The specimen measured approximately 15 × 12 cm and weighed 462 g. Blood loss was controlled, and two units of packed red blood cells were transfused.

**Figure 3.**
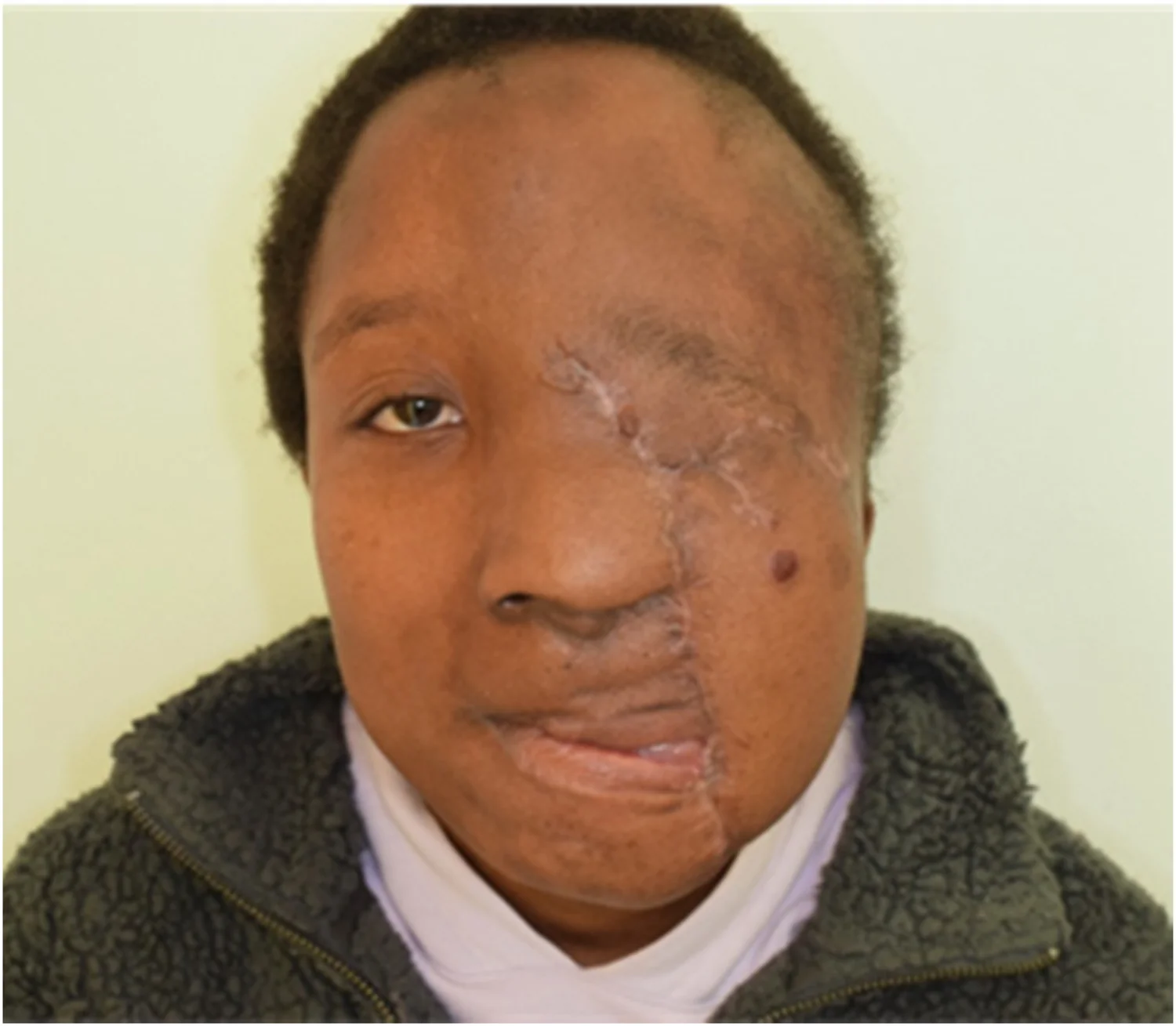
Three weeks postoperatively, after wide local excision and immediate reconstruction with an anterolateral thigh free flap, showing improved facial contour and early resolution of postoperative swelling.

Immediate reconstruction was performed with a 12 × 10 cm anterolateral thigh free flap anastomosed to the facial vessels. Static suspension sutures from the flap dermis to the zygomatic arch and temporalis fascia supported the oral commissure and cheek. The donor site was closed primarily. Total operative time was 8 h. The patient was extubated postoperatively and monitored for 24 h in intensive care. The flap remained viable, the drain was removed on day 3, and she was discharged on day 6. At 3 weeks, facial contour and symmetry were markedly improved, oral competence was preserved, and the patient was highly satisfied despite expected ipsilateral facial paralysis (Figure 3). Histopathology confirmed benign PNF without malignant transformation. At 22 months, she had undergone minor excision of a small localized mucosal recurrence identified at 9 months, with no further recurrence.

## Discussion

This case demonstrates why radical one-stage management may be appropriate for selected giant facial PNFs in LMIC settings. Subtotal excision may seem safer because it preserves structures and avoids a large defect; however, residual PNF is infiltrative, vascular, and prone to regrowth.^3,5^ In this context, the question is not whether radical surgery is aggressive, but whether repeated incomplete surgery is truly conservative.

The senior author's cumulative experience with 34 giant facial PNFs over two decades is not presented here as a formal cohort, but it is central to the judgement described. Earlier reliance on staged debulking was associated with recurrent growth, progressive distortion, repeated admissions, and increasingly difficult operative fields. The strategy evolved toward single-stage macroscopic clearance when the tumor was anatomically resectable and the patient could tolerate major reconstruction. In that experience, radical excision has not eliminated recurrence, but it has tended to change recurrence from a massive, deforming problem into a localized and more manageable one, as occurred in the present patient. This distinction is especially important where follow-up is uncertain and return for multiple operations may be impossible.

The most important technical decision was to pursue macroscopic clearance rather than preserve grossly involved skin, mucosa, parotid tissue, and facial nerve branches. This is not oncologic margin surgery; microscopic clearance is often impossible because PNFs grow diffusely along nerves and tissue planes. The operative goal is removal of all visible and palpable disease while accepting reconstructable deficits. Attempts to preserve involved structures may prolong surgery, increase haemorrhage, and leave the patient with predictable major recurrence.

Immediate vascularized reconstruction converts a destabilizing hemifacial defect into a stable contour and protects exposed structures. Where dynamic facial reanimation is unavailable, static suspension offers a durable, low-maintenance solution that improves resting symmetry and oral competence.^6^ A single definitive admission may also reduce cumulative exposure to anaesthesia, transfusion, travel, family disruption, and scarce inpatient resources. Although MEK inhibitors have expanded treatment options for selected inoperable PNFs, they require sustained access, monitoring, and cost structures that remain unrealistic for many LMIC patients.^7^ For this patient, surgery provided immediate rehabilitation, early discharge, and a small manageable recurrence rather than another cycle of major regrowth.

## Conclusion

In selected patients with giant recurrent facial PNF in resource-limited settings, single-stage radical excision with immediate free flap reconstruction can provide durable tumor control, early discharge, and major aesthetic and psychosocial benefit. Functional sacrifice may be justified when involved structures are unlikely to recover, and their preservation would leave progressive disease.
